# Supplementary material for: Complex‐centric proteome profiling by SEC‐SWATH‐MS
Source: Mol Syst Biol. 2019 Jan 14;15(1):e8438. doi: 10.15252/msb.20188438 (PMC6346213; doi:10.15252/msb.20188438)
Supplement: Supplementary file 8 — Dataset EV7 [file MSB-15-e8438-s008.zip › feature_plots_string/Q9Y584.pdf]

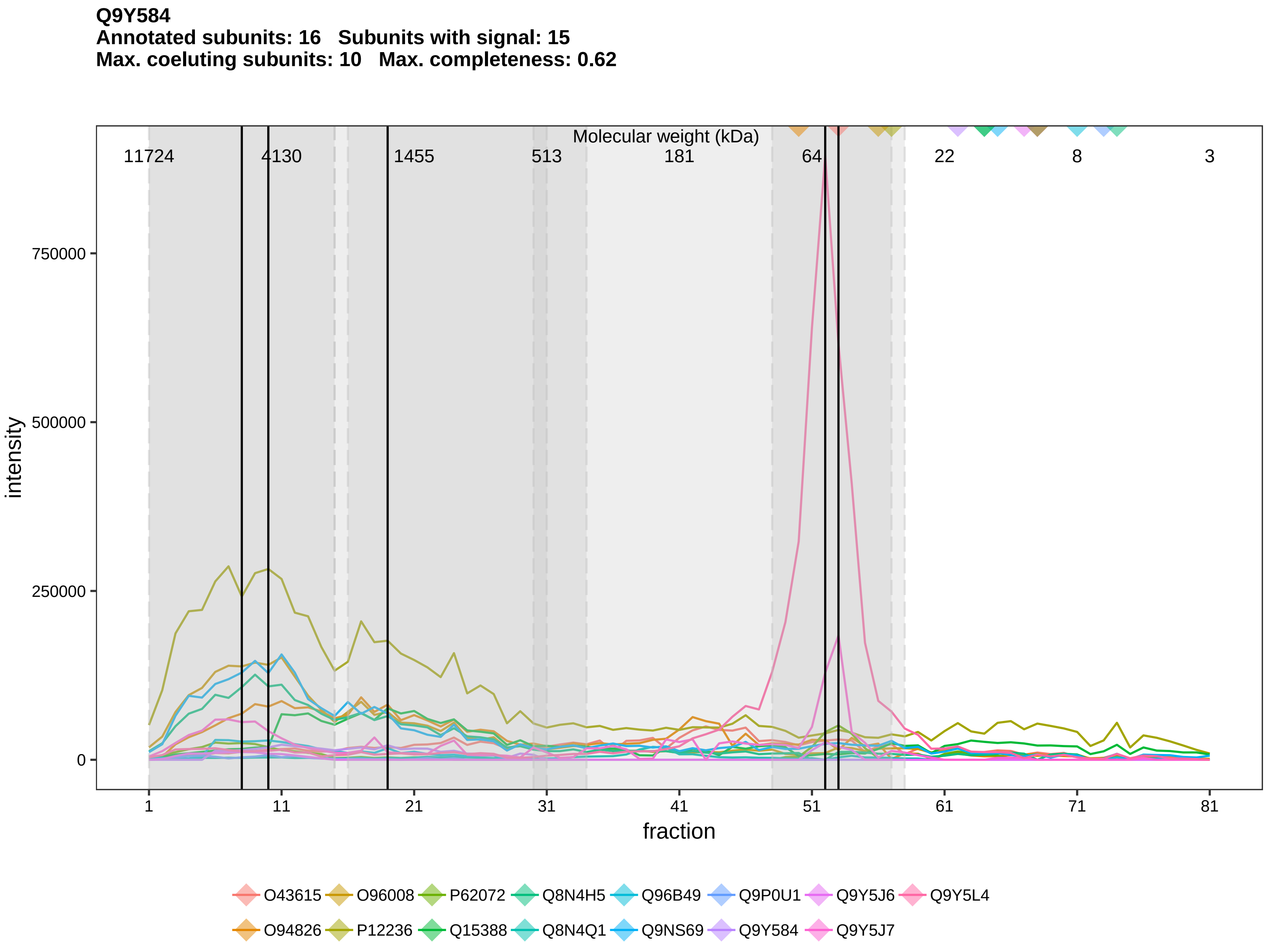
Q9Y584
Annotated subunits: 16 Subunits with signal: 15
Max. coeluting subunits: 10 Max. completeness: 0.62
Molecular weight (kDa)
11724
4130
1455
513
181
64
22
8
3
intensity
750000
500000
250000
0
fraction
1
11
21
31
41
51
61
71
81
O43615
O96008
P62072
Q8N4H5
Q96B49
Q9P0U1
Q9Y5J6
Q9Y5L4
O94826
P12236
Q15388
Q8N4Q1
Q9NS69
Q9Y584
Q9Y5J7
